# PlanktoVision – an automated analysis system for the identification of phytoplankton

**DOI:** 10.1186/1471-2105-14-115

**Published:** 2013-03-27

**Authors:** Katja Schulze, Ulrich M Tillich, Thomas Dandekar, Marcus Frohme

**Affiliations:** 1 Biotechnology and Functional Genomics, Technical University of Applied Sciences, Bahnhofstraße, Wildau, 15745, Germany; 2 Bioinformatics, University of Wuerzburg, Biocenter, Am Hubland, Wuerzburg, 97074, Germany

## Abstract

**Background:**

Phytoplankton communities are often used as a marker for the determination of fresh water quality. The routine analysis, however, is very time consuming and expensive as it is carried out manually by trained personnel. The goal of this work is to develop a system for an automated analysis.

**Results:**

A novel open source system for the automated recognition of phytoplankton by the use of microscopy and image analysis was developed. It integrates the segmentation of the organisms from the background, the calculation of a large range of features, and a neural network for the classification of imaged organisms into different groups of plankton taxa. The analysis of samples containing 10 different taxa showed an average recognition rate of 94.7% and an average error rate of 5.5%. The presented system has a flexible framework which easily allows expanding it to include additional taxa in the future.

**Conclusions:**

The implemented automated microscopy and the new open source image analysis system - PlanktoVision - showed classification results that were comparable or better than existing systems and the exclusion of non-plankton particles could be greatly improved. The software package is published as free software and is available to anyone to help make the analysis of water quality more reproducible and cost effective.

## Background

The composition of phytoplankton communities is dependent on different ecological and toxicological factors and the analysis of these communities entails the monitoring of water quality which is prescribed by the European Water Framework Directive [1,2]. However, this approach is time consuming since the organisms have to be registered individually and, due to the high morphological complexity of phytoplankton, most of the routine analysis is still done by hand and microscope. Also reproducibility between different scientists and even for the same person is relatively low and reduces the credibility of the retrieved data [3].

To overcome these problems, automation of phytoplankton analysis seems to be a good alternative. It should speed up the process, make it more transparent and reproducible, and therefore improve the effectiveness of phytoplankton identification for water monitoring and protection. Because of these advantages, different systems for an automated analysis of phytoplankton communities have been developed.

The use of flow cytometers allows a fast analysis of different plankton groups [4] and removes the need to preserve the sample [5]. Yet the strongest drawback of this method is that particles must be differentiated based on the optical characteristics as seen by the photomultiplier tube (e.g. light scattering and fluorescent features) resulting in poor species resolution, far below microscopic methods (especially for nano- and microplankton) [5,6]. Another noteworthy approach is the investigation of the whole phytoplankton community in parallel via a metagenomic analysis. Since this method is based on genomic data, it has great potential for high taxonomic resolution [7]. Unfortunately, the costs are still too high to be feasible for a routine analysis, though this is expected to change in the future with the rapid advancements in this field. This also applies to some extent to modern barcoding methods based on the sequence and structure of genetic markers such as ribosomal RNA [8]. Quantification via metagenomic analysis or related barcoding methods may also be difficult as DNA or RNA molecules and not cells are counted and the number of genomes of each organism (which varies not only depending on species, but also on growth phase) has to be known. Finally, the identification of a DNA needs a previously sequenced reference genome, i.e. one can only properly identify species which have been studied beforehand.

Since taxonomy of plankton is based on morphological differences, various systems using digital image analysis (scanner, flow systems, video systems) can be found [9]. Yet, these systems are mostly developed for the analysis of zooplankton resulting in a resolution too low for an accurate differentiation of phytoplankton. Due to their higher resolution, microscopic systems seem to be more adequate for the analysis of this plankton subclass. Additionally, microscopes are currently used for the established procedures for plankton analysis, thereby allowing first an adaptation and then an easy comparison of both systems. One reported automated microscopic system is PLASA, which was developed with the goal of classifying different phytoplankton organisms with the use of automated microscopy and image analysis for an ecotoxicological microcosm study [10]. To allow a better differentiation between different phytoplankton taxa and between phytoplankton and other objects in the sample (zooplankton, detritus and inorganic particles) fluorescence imaging for phycoerythrin and chlorophyll was integrated into the system. The software was written in IDL, a proprietary programming language requiring a commercial license to legally run the respective programs, which potentially hindered the development and adoption of this system. Additionally, it has been reported to only be able to differentiate between few taxa, and has seen no further development since it was first published in 2006.

In this paper we describe a novel system using automated microscopy and image analysis for the automated identification of phytoplankton for monitoring freshwater quality. Like PLASA, the microscopic system uses fluorescence imaging, for a better discrimination. The chlorophyll filter set used in PLASA was improved and in addition to the filter for phycocyanin, a new filter set for phycoerythrin was also integrated into the analysis. Additionally, a new method, where different focal levels are integrated into one image during the microscopy (Quick Full Focus images), was used. One of the goals was to develop open source software that is available for a broad range of research. Therefore, the image analysis was written as a plugin for ImageJ, which is a free and open source project written in Java [11]. This allows the use of the software on almost any operating system without costs. Since the code for the plugin is also licensed as free software anyone can adapt and expand the system.

PlanktoVision was specifically developed to improve water quality analysis. In order to make this possible, we so far focused on some of the most important taxa (indicator taxa) as defined by the harmonized taxa list for phytoplankton in Germany. This list is based on a nation-wide study with the goal to standardize phytoplankton analysis to make it more reproducible [12].

For the automated classification the use of neural networks was examined. Neural networks consist of artificial neurons, resembling the properties of biological neurons. These neurons are equations connected in different layers and allow complex classification tasks.

In the following, PlanktoVision and the used microscopic system are described. Initial results for the classifications of 10 different indicator taxa using a neural network are discussed.

## Results

### Automated image acquisition

Images within the scanning path showed a consistent quality (e.g. no change in the illumination or background color) and no significant changes in the characteristics of the sample could be observed during the microscopy (data not shown). Because of an uneven bottom of the sedimentation chamber an auto focus function had to be used for every position resulting in a longer image acquisition time.

Since typical bright field images only integrate one focal plane into the image, parts of some organisms with a larger volume were, however, still blurred, which occurred more obviously in the mixed samples. In the Quick Full Focus images (QFF images), which integrate different focal planes into one image, all organisms could be imaged completely (without blurred parts) in the mono culture samples, as well as in the mixed sample (see example images in Additional file 1). With this method, however, it is essential to avoid vibration during the imaging process to prevent the organisms from moving and creating artefacts in the image. For the fluorescence imaging it could be observed that the short exposure of a single position already caused bleaching effects that reached further than the immediate area of the taken image. To keep the bleaching on the different recording positions to a minimum the distance between them was set to 500 μm. Additionally, the gain for the fluorescence images was set as high as possible (without affecting the subsequent image analysis because of high noise in the image) to keep exposure times as short as possible. After this change, the characteristics of the analyzed samples remained stable during the imaging procedure.

### Image processing

#### Adaptation of ImageJ

All image processing and classification tasks were performed with ImageJ since it already integrates many functions for the work with microscopic images. Additionally, ImageJ is an open source project that can be easily extended. Nevertheless, it is not primarily intended to be used for complex classification tasks as presented in this work. To allow this, ImageJ’s core functionality had to be expanded. This includes the integration of a region growing segmentation algorithm and the calculation of additional features (see Methods). ImageJ is also unable to perform classification tasks by itself. To allow this, the classification API Encog [13] was integrated into the system.

#### Segmentation

**Figure 1:**
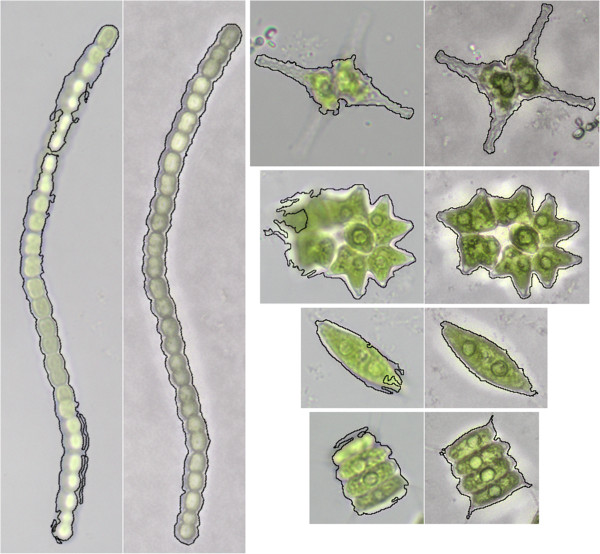
**Exemplary segmentation results for bright field and Quick Full Focus images.** The images show the segmentation of taxa that have a certain three dimensional structure. The part of the image which was segmented from the background is marked by a black line. The segmentation is shown for a bright field image (on the left side) and an according Quick Full Focus image (on the right side).

In this step of the image processing all particles in an image are separated from the background and registered individually. Comparing both types of images (typical bright field and QFF images) the separation of the single organisms from the background was better for the QFF images (Figure 1). Since they integrate different focal planes into one image most of the organisms could be properly segmented and an accurate feature calculation (and classification process) was possible. For the bright field images a good segmentation was only possible when the organism was imaged at, or near to its focal point. However, it was often impossible to obtain images with all organisms in focus. Some organisms have three dimensional structures which can not be focused simultaneously and not all organisms occupy the same focal level when sedimented. When the segmentation of organisms that were not in focus was possible it still resulted in an inaccurate feature calculation and a poor classification process. Based on these results only QFF images were used for the following training and testing.

#### Selected features & classification

After the segmentation, different features are calculated (see Method section “Feature calculation” for a full overview) that describe the characteristics of the particles. These features are then used in the classification step to differentiate the plankton species. It has to be noted that not all calculated features are used for the classification that is presented within this manuscript as the use of some features reduced the classification results with our species selection (data not shown). However, these features could be useful to differentiate between a different set of taxa. The features selected for our work are listed in the section below.

The classification procedure was divided into two steps. First a neural network was trained to separate plankton particles from non-plankton particles; the latter exhibited a great range of texture, size and shape and interfered with the actual classification of the plankton. Since no non-plankton particles showed any fluorescence and all were mostly transparent or brown, features for color (hu histogram), fluorescence (mean brightness in the Roi of phycoerythrin, phycocyanin and chlorophyll fluorescence), area and circularity showed good classification results with the mixed test sample (Table 1).

**Table 1:** Confusion matrix for the classification results

	**PlanktoVision**													
	**det**	**ukw**	**1**	**2**	**3**	**4**	**5**	**6**	**7**	**8**	**9**	**10**	**% recognition**	**% false positive**
**det**	**11061**	0	0	1	0	0	0	0	0	0	0	0	99.99	0.00
**ukw**	0	**371**	0	2	0	4	3	0	7	3	0	13	92.06	8.09
**1**	0	3	**91**	0	0	0	0	0	1	0	1	0	94.79	6.59
**2**	0	5	3	**182**	0	0	0	0	2	0	0	1	94.30	3.85
**3**	0	1	0	0	**57**	1	1	0	0	0	0	0	95.00	0.00
**4**	0	0	0	2	0	**115**	0	0	0	0	0	0	98.29	8.70
**5**	0	1	0	0	0	0	**124**	2	8	3	0	0	89.86	4.03
**6**	0	7	0	2	0	0	0	**94**	0	0	1	0	90.38	2.13
**7**	0	2	1	0	0	0	0	0	**253**	2	1	2	96.93	7.51
**8**	0	0	0	0	0	0	0	0	0	**59**	3	0	95.16	13.56
**9**	0	1	0	0	0	5	0	0	0	0	**127**	0	95.49	4.72
**10**	0	10	2	0	0	0	1	0	1	0	0	**240**	94.49	6.25
**Ø**													94.73	5.45

The data were classified into detritus (det), unknown plankton organism (ukw), *Cyclotella* (1), *Anabeana* (2), *Chlorogonium* (3), *Cryptomonas* (4), *Desmodesmus* (5), *Staurastrum* (6), *Botryococcus* (7), *Pediastrum* (8), *Trachelomonas* (9) and *Crucigenia* (10). The test set included images of 4 different samples that were independently prepared, imaged and analyzed on different days to prevent the selection of an over fitted classifier. The results of PlanktoVision (columns) were compared to a manual classification (rows). Correctly classified results are shown in bold.

In a second step, another separate network was trained for the actual differentiation between the plankton taxa. Best results for the mixed test sample could be achieved with 2 hidden layers (first hidden layer 50 neurons, second hidden layer 30 neurons) and features for the texture inside of the organism (local binary pattern, image moment 1), shape (eliptic fourier descriptor 2–13, circularity, roundness, solidity, minimum feret and perimeter), size (area), and pigmentation of the organism (color via hu histogram; single photo pigments via fluorescence).

The classification of the test sample showed an average recognition rate of 94.7% ranging from 89.9–99.9%. The rate of false positive particles showed an average of 5.5% (ranging from 0–13.6%) and was mainly caused by occluded or aggregated organisms, organisms that did not show the typical morphological features, incorrectly segmented particles or particles that were out of the focus range.

## Discussion

**Figure 2:**
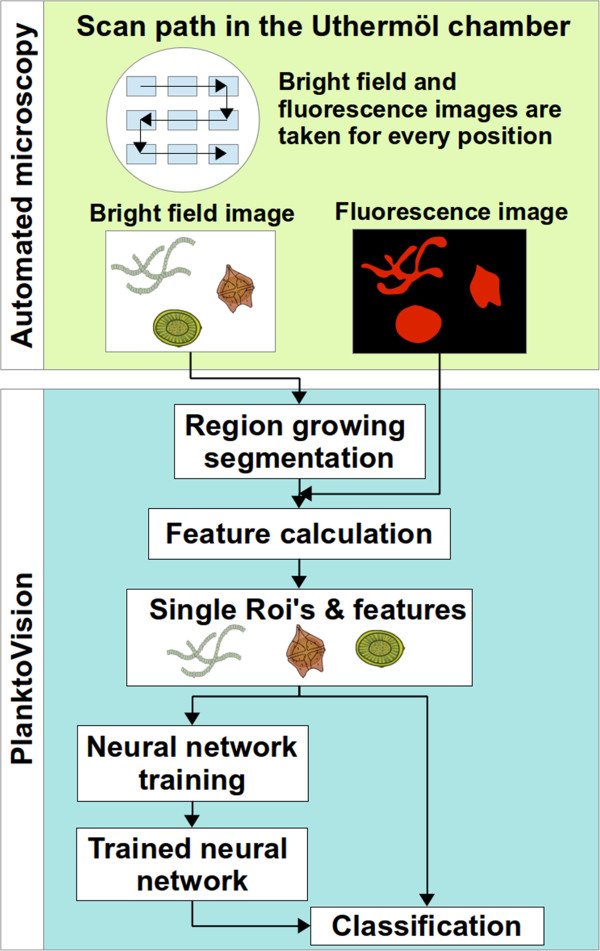
**Overview of the plankton analysis.** During the automated microscopy bright field and fluorescence pictures are taken for different positions in the Utermöhl chamber. For the image analysis all particles are segmented from the background of the bright field image and features are calculated. After manual sorting, the segmented images can be used to train a neural network which is then able to classify new images according to taxon.

We present here a system for the automated identification of phytoplankton using microscopy and computer-based image recognition in regard to water quality analysis (see Figure 2 for an overview). It integrates methods already described [10,14] as well as new approaches into the analysis. The procedure performed by the system can be divided into the two general steps: automated imaging of the water sample and the following image processing and recognition.

The sample was sedimented in an Utermöhl chamber and automatically scanned in the microscope. To enable a precise analysis of the samples, different images (bright field images and autofluorescence of the organisms) were taken for each position during the scanning process. Quick Full Focus images (QFF), which integrate different focal planes into one image, were newly implemented in a plankton classification system. This has two distinct benefits compared to regular bright field images. On the one hand plankton organisms with a large expansion over the focal plane can be imaged in total, even when using a great magnification, improving the accuracy of the following image processing and recognition (segmentation, feature calculation and classification). On the other hand, the loss of whole groups of organisms, which are mostly located at a higher focal point above other sedimented organisms (due to spikes that are attached around the organism or transparent capsules into which an organism is embedded), can be reduced. This results in a smaller overall systematic error for the system (data not shown).

Since the used Keyence software is proprietary, it was not possible to determine exactly how QFF images are generated. This may complicate the adaption of PlanktoVision to other microscope systems, which probably have similar but not necessarily identical functions available. To avoid these problems and make methods available to more people it is advisable to use open source solutions for the control of microscopes (if available). One possible solution would be the use of the micro manager software [15]. Micro manager allows the recording of z-stacks and since it is based on ImageJ, existing or newly created plugins for a z-stack projection could be easily integrated. The currently supported hardware of Micro manager includes single components as well as fully motorized microscopes from leading manufacturers. Unfortunately, micro manager does not support the hardware currently used for the development of PlanktoVision and therefore could not be integrated.

In addition to the fluorescence of chlorophyll and phycoerythrin, which was already recorded by the PLASA system, the imaging of the phycocyanin fluorescence was newly integrated in the imaging process. Unlike as described for PLASA [14], we used a chlorophyll filter which is able to excite chlorophyll a and chlorophyll b simultaneously so that both could be included into the analysis. The filter set used for PLASA has been reported not to induce chlorophyll fluorescence for some species during the experiments. It only excites chlorophyll b, which is not present in the photo system of many phytoplankton species [16].

The image processing itself can be divided into three distinct steps: (1) segmentation of the organisms from the background; (2) calculation of features that describe the segmented particle in a more defined way; and (3) classification of the different groups of organisms based on the calculated features.

For the segmentation a region growing approach was chosen [17]. Compared to a histogram-based thresholding, where the whole image influences the segmentation, it enables a more robust segmentation of very different organism. This is possible since only the background region is included into the calculations for the segmentation and the amount and type of the organisms in the analyzed image has no influence. Edge detection (in the brightness channel) was integrated into the region growing, to allow a good segmentation of transparent organisms and organisms that include transparent parts. Because of this, the segmentation was only possible for organisms that were imaged at their focal point, since edges are only detectable at sharp intensity differences in the image. This, however, is not a drawback, as the segmentation of organisms that are out of focus results in a reduced accuracy, or even erroneous feature calculation and has an overall negative impact on the analysis. Therefore the positive effects of the edge detection vastly outweigh its disadvantages and we conclude for this application that the combined approach of edge detection and region growing is superior to a region growing without previous edge detection.

For the feature calculation, new features (especially for the texture description) as well as those previously reported (e. g. in PLASA) were used. Despite the fact that most factors for illumination were set to fixed values and the image as well as the rotation of the organisms was corrected before classification, it was noted that few features, which are not invariant to those factors, were less suitable for classification. These features include statistics of the gray level co-occurrence matrix as well as normalized brightness and saturation of the organisms. Also features that are influenced strongly by slight variations in shape (such as fourier descriptor 12–29, or the aspect ratio) seem to deteriorate the results since the shape varies to differing extents with the different organisms.

The classification of the organisms described was done by using neural networks. For the differentiation of all taxa one network with 12 output possibilities (ten for the respective taxa, one for detritus, and one for unknown particles) was chosen and trained with one pre-selected set of features. Another possibility would be to use one network for every organism with a feature set optimized and specifically selected for this organism. In this case, classification would be done by going through every network and checking if the particle belongs to one class or not. However, training and feature selection for the different networks would be more complex and every network would have to be checked and (when needed) adapted to each new organisms integrated into the system. Since the classification with one network showed a good performance this approach was preferred due to its simplicity and expandability.

Despite the average classification rate of ~ 95%, an over-training of the network can be excluded as the image test set contained images from independent samples and all samples showed good classification results without bias towards a wrong class. A comparison of the classification rate to other published systems is difficult since there are differences in the number of classes, as well as varying similarities of the organisms that should be differentiated. Additionally, most of the reported systems are used for differentiation of marine plankton.

Culverhouse [18], however, made a comparison of reported systems. The classification results are summarized as followed: ADIAC (37 taxa, 75–90% recognition), which is a system for the automated analysis of diatom slides [19]; Zooscan (29 groups, 75–85% recognition), which is a platform for the analysis of mesozooplankton where samples are imaged with a water-proof scanner [20,21]; SIPPER (5 groups, recognition of 75–90%) [22] and VPR (7 groups, 72% recognition) [23], which both are system for the analysis of mesozooplankton, where the organisms are imaged during the sampling; DiCANN (3–23 species, 70–87% recognition), which is a system for the classification of dinoflagellates [24]; Cytosense (30 groups, 91% recognition), which is a flow cytometric approach [25].

The average recognition rate of PLASA (~ 94% for 5 classes) was above the recognition rate of the previously mentioned systems and is comparable to the rate achieved by PlanktoVision (~ 95% for 10 classes). Nevertheless, PlanktoVision showed a much smaller rate of false positives (~ 6%) when compared to other reported results (PLASA 20%; VPR 55% [23], Zooscan 26% [21]). For all the mentioned systems the false positives were mostly caused by a wrong classification of non-plankton particles and unidentified objects.

In PlanktoVision this cause of errors could be drastically reduced by the use of the adapted chlorophyll fluorescence filter which allowed a very good exclusion of non-planktonic particles from the analysis with an error rate < 1%. Additionally, “unknown” particles (e.g. particles that were wrongly imaged and/or segmented and organisms that did not show the typical morphological features or were occluded/aggregated with other particles or organisms) could be identified with ~ 92% accuracy during the analysis, despite the fact that they showed a great range of shape, color and fluorescence. Additionally, the system allowed a good classification of taxa that show similarities in their morphology (for example *Cryptomonas* and *Trachelomonas,* with a round and quite similar shape) as well as a good differentiation of taxa that show varying morphology within their class (e.g. *Botryococcus* and *Anabeana* have one basic cell shape, but the number of cells in an aggregate changes while growing).

Comparing the average classification rate of ~ 95% achieved by PlanktoVision to human accuracy, the results are in the same range as those reported for routinely engaged personnel (84–95% accuracy) and notably better than those for trained but not routinely engaged personnel (67–83% self consistency and 43% consensus between trained personnel) [3]. Here it has to be stated that an analysis of a sample by a human generally does not provide a reproducible error rate - despite an automated system.

## Conclusions

The implemented automated microscopy and the new open source image analysis system -PlanktoVision- allowed a good differentiation of the presented test set consisting of 10 different phytoplankton taxa. The classification results were comparable or better than existing systems and the false positive rate could vastly be improved over reported results due to a better exclusion of non-plankton particles and unidentified objects. The image analysis was developed as an open source system in order to make it available for many researches and thereby help to make the analysis of water quality more reproducible. For future work, more taxa should be integrated into the analysis to allow the generation of more significant results in regard to the water quality analysis of real phytoplankton samples. However, the chosen methods for the image processing might have to be revised to test if they are still sufficient, or if they will have to be further extended (e.g. the integration of other feature calculation methods). Additionally, the choice of the neural network structure might have to be reexamined for the differentiation of a larger number of taxa.

## Methods

### Strains

**Figure 3:**
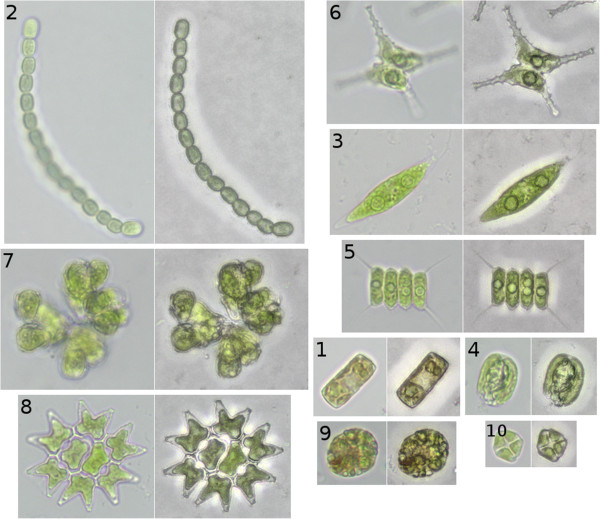
**Bright field microscopic images and Quick Full Focus images of the analyzed taxa.** For every pair of images the left side shows the bright field image and the right side shows the Quick Full Focus image. The taxa are: *Cyclotella* (**1**), *Anabeana* (**2**), *Chlorogonium* (**3**), *Cryptomonas* (**4**), *Desmodesmus* (**5**), *Staurastrum* (**6**), *Botryococcus* (**7**), *Pediastrum* (**8**), *Trachelomonas* (**9**) and *Crucigenia* (**10**).

For the training and testing of the system 10 different taxa (Table 2) from mono-cultures were fixed with 1% paraformaladehyde to preserve fluorescence characteristics. The morphological characteristics can be seen in Figure 3.

**Table 2:** Used taxa for the training and testing of PlanktoVision

**Strain**	**Origin**
*Cyclotella meneghiniana*	SAG 2136
*Anabaena sp.*	CBT 149
*Chlorogonium elongatum*	SAG 31.98
*Cryptomonas ovata*	SAG 979-3
*Desmodesmus perforatus*	Isolated by U. Mischke from the “Müggelsee” lake, Berlin
*Staurastrum tetracerum*	SAG 7.94
*Botryococcus braunii*	SAG 807-1
*Pediastrum duplex*	SAG 28.83
*Trachelomonas volvocina*	SAG 1283-4
*Crucigenia tetrapedia*	SAG 9.81

### Sedimentation

For the image acquisition the fixed samples were sedimented according to the Utermöhl method [26]. The sample volume was chosen so that the sedimented cells did not occlude each other on the bottom of the chamber. To obtain training images for the different taxa the mono-cultures were sedimented separately. To obtain test images, different mixed samples consisting of all taxa were sedimented.

### Automated microscopy

For the image acquisition the computer controlled inverse Keyence BZ 9000 fluorescence microscope was used. The microscope was equipped with the following fluorescence filter sets: chlorophyll a & b (excitation: 435/40 nm; beam splitter: 510 nm; emission: 515 nm long-pass), phycoerythrin (excitation: 543/22 nm; beam splitter: 562 nm; emission: 593/40 nm) and phycocyanin (excitation: 600/37 nm; beam splitter: 625 nm; emission: 655/40 nm). To allow a fully automated analysis of the samples, a mouse recorder software was used to program a scanning routine, where images for 80 different positions were taken within a predefined rectangular area. Before starting the image acquisition, lighting, aperture stop, and exposure time for the fluorescence imaging were adjusted to predefined values (bright field: exposure time 1/28s, aperture 65% open, light intensity 84% ; fluorescence: exposure time 1/4,5s and gain 18dB). Additionally, a white balance was performed and an image without the sample was stored for image correction. During the scanning the auto focus function of the Keyence software was started for every position (since the glass slide in the Utermöhl chamber is not necessarily completely even). Afterwards, five different images were taken with an 60× objective: A normal bright field image, a Quick Full Focus image (QFF) that integrates 35 different focal levels above and 23 focal levels below the auto focused image into a single image, and fluorescence images addressing the absorption/emission spectra of chlorophyll, phycoerythrin and phycocyanin.

### Image processing

The image processing routines were written in Java as open source plugins for ImageJ. They were separated into the four plugins PVsegment, PVtrainer, PVclassifier and PVanalysis. PVsegment implements the segmentation of the particles from the background and the calculation of different features. PVtrainer includes the training process and PVclassifier can be used for the classification using the trained classifier trained in PVtrainer. Additional PVanalysis integrates the segmentation, feature calculation and classification into one plugin and allows a fully automated analysis of the images.

#### Image preprocessing

Different factors during microscopy (for example changes in the light source or the optical system) can influence the brightness and color within the taken image. To minimize these effects an image without the sample was taken before every scan and used in a preprocessing step for a correction of the original images. This was done by using the divide function of the CaluclatorPlus from ImageJ with the following formula: I_cor_ = (I_sample_ / I_lc_) * mean_lc_ where I is the pixel brightness of the respective image (cor - corrected image; s - image of the sample, lc - image without the sample) and mean_lc_ is the global mean of the brightness.

#### Segmentation

A region growing approach was chosen to separate the organisms from the background of the microscopic image. To enable a better segmentation of transparent organisms an edge detection using the standard Sobel operator and contrast adjustment (Histogram normalization using 0.4% of the saturated pixel (ImageJ function “Enhance Contrast”)) was performed as preprocessing. The basic principle of the method is to start with pixels lying in the background as seed points and add adjacent pixels that fit a defined background criterion allowing the segmentation between background and organisms. To find correct seed points of the background, the watershed segmentation of the MaximumFinder in ImageJ was used resulting in a rough segmentation of the image into different areas. For every area the mode pixel value was determined (i.e. the pixel value that appears most often). Since in this type of images the background is the largest even part of any given image, the largest area with the same mode value was chosen as being the background. For the region growing segmentation all pixels with the mode value within this area were then set as seed points. Afterwards, the region growing is started by adding adjacent pixel that fit the background criterion. Different background criteria were tested. Best results could be retrieved with the standard deviation of the brightness of all pixels already marked as background multiplied by 10.To register the different segmented particles as regions of interest (Roi) the Particle Analyzer function is then used [27]. Particles smaller than 100 pixels (~3 μm^2^) and particles touching the edge of the image were excluded from further analysis.

#### Feature calculation

To enable a classification of the registered regions in the image a set of different kinds of features, which describe these areas in a more defined (and less complex) way, were calculated. To reduce the influence of the rotation of the particles the angle between the primary axis of a fitted ellipse and the x-axis of the image was used to rotate the particle to an angle of zero. Basic features were recorded with the measurement function of ImageJ. For more complex features, available plugins were integrated into the system. This includes elliptic fourier descriptors of the contour [28], statistics of a gray level co-occurrence matrix (glmc) of the Roi [29,30], a directionality histogram [31] and different image moments [32]. A symmetry measurement [33], rotation invariant local binary patterns [34] and the extraction of fluorescent features was newly integrated into the system. All features are listed in more detail in Additional file 2. After the calculation of all features was completed the Roi’s and features for the image were saved. For the creation of the training set every Roi was saved as a single image to enable the sorting into different classes.

### Creation of training- and test-data

For the training set, all mono cultures were chemically fixed and images were taken under the microscope with the previously described automated procedure. The pictures were segmented and features were calculated for every segmented particle. After the segmentation, particles were sorted by hand into different categories: detritus, incorrectly segmented or unknown plankton organisms, or one of 10 groups for the particular taxon. The size of the training data was around 600 images per class.

For the creation of the test set, images for a mixture of all 10 plankton taxa were used and processed in the same way as the images for the trainings set. In order to prevent the selection of an over-trained classifier, which would only correctly work on the image set used for training, four different samples were independently prepared, imaged and analyzed on different days.

### Training and classifier

For the classification of the different plankton taxa a neural network was used. Encog, a java framework for neural networks, was integrated into ImageJ [13]. The network type used was a simple feed forward network with an Elliott activation function [35] since this showed the best training efficiency.

For the selection of the most suitable features, all available features were used for an initial training. Based on these results the significance of every input neuron was determined with a function integrated in the used API and the best suited features selected. Additionally, an empiric testing was performed where different features were subsequently added to the network and the impact for a better classification was determined with the test set (the selected sub-set of features is listed under the results section “Selected features & classification”).

Whenever the network was modified (e.g. different feature set or output count) one training was performed with an incremental pruning to determine the best number of hidden layers and of the neurons used in these layers. To start the training, all data of the training set was (when needed) normalized to ranges between -1 and 1. The resilient propagation algorithm was chosen as training method [36,37]. Training was performed to an error rate of 0%, or a maximum of 3.000 iterations. After the training was finished, the neural network and the rules for the normalization of the data were saved and used for all further analyses. For testing of the classifier the data of the test set was normalized according to the saved normalization rules and classified with the trained neural network. To allow a verification of the results, the single images of the Roi’s were stored in folders according to their classified class and compared to the results of a classification made by hand.

### Availability

The ImageJ plugins and a data set supporting the results of this article are available in the github repository through https://github.com/KatjaSchulze/PlanktoVision. The software is licensed under the GNU General Public License, Version 3.

## Competing interests

The authors declare that they have no competing interests.

## Authors’ contributions

KS designed and implemented the software package, carried out the microcopy, performed the analysis of the test data and wrote the manuscript. UMT helped with the design of the software package, contributed to the writing of the manuscript and helped to cultivate the strains used for testing of the system. TD contributed to the design of the system and helped to draft the manuscript. MF supervised the research, provided laboratory facilities and contributed to the writing of the manuscript. All authors read and approved the final manuscript.

## Supplementary Material

Additional file 1 Comparison of bright field and Quick Full Focus images for the same position. Click here for file

Additional file 2 Description of the used Features in PlanktoVision. Click here for file
