# Supplementary figures and images for: PlanktoVision – an automated analysis system for the identification of phytoplankton
Source: BMC Bioinformatics. 2013 Mar 27;14:115. doi: 10.1186/1471-2105-14-115 (PMC3636010; doi:10.1186/1471-2105-14-115)

**Bright field image**

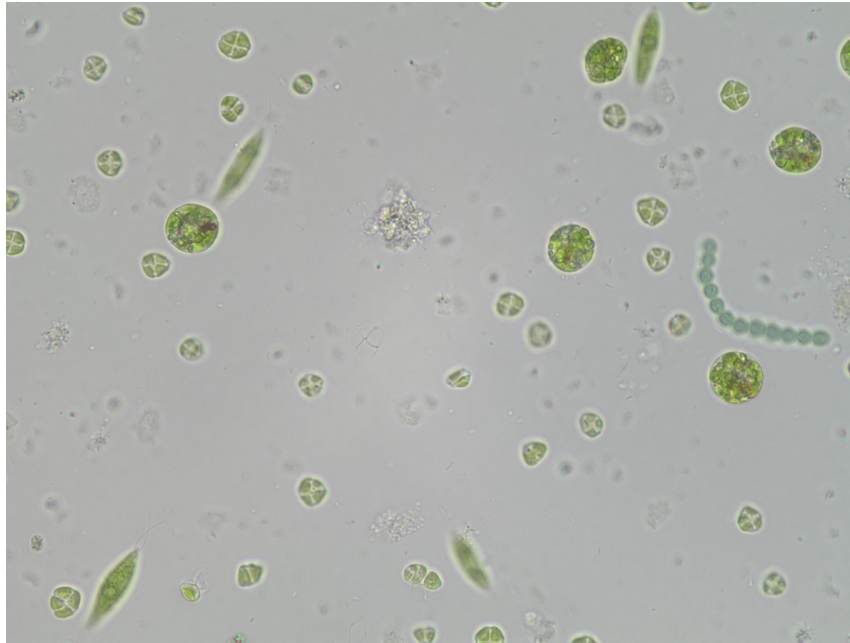

**Quick Full Focus image**

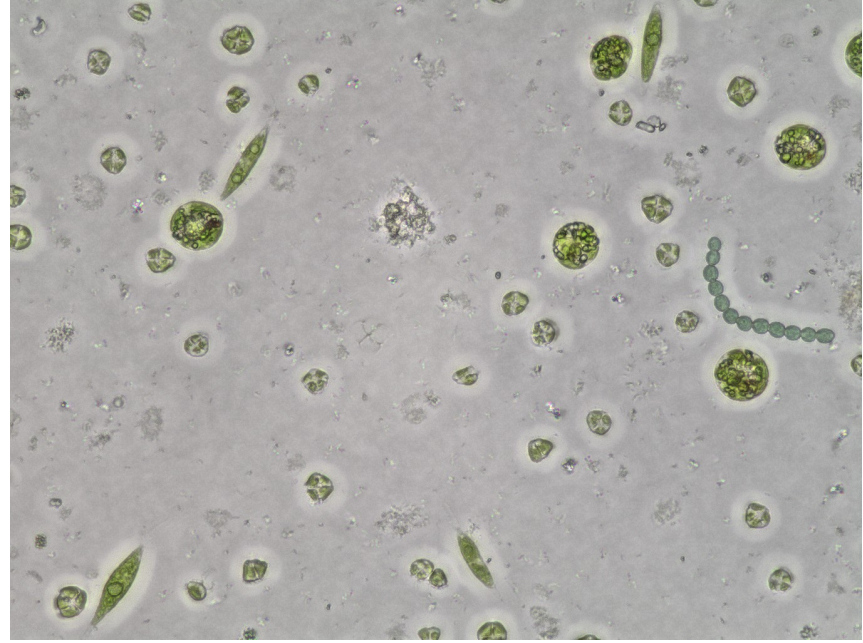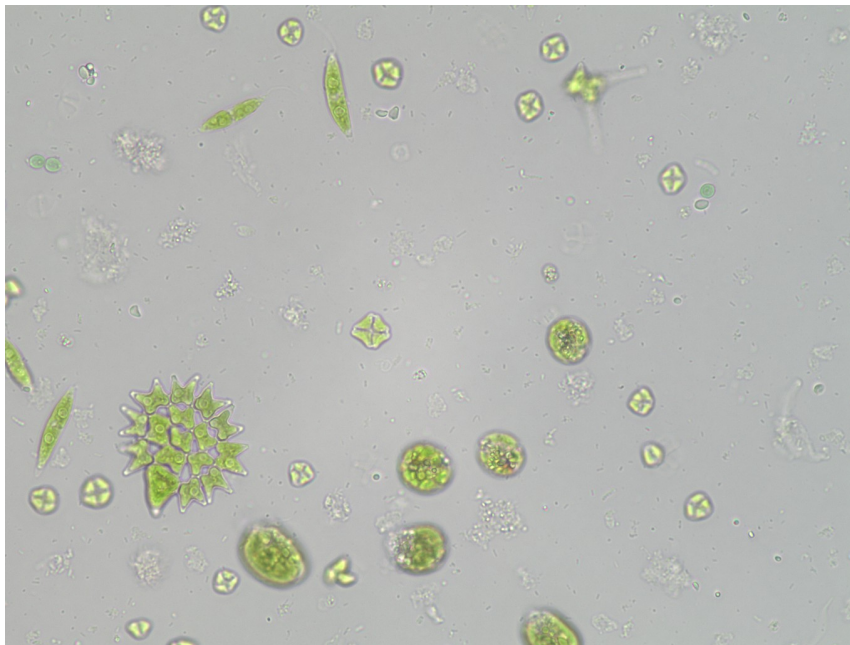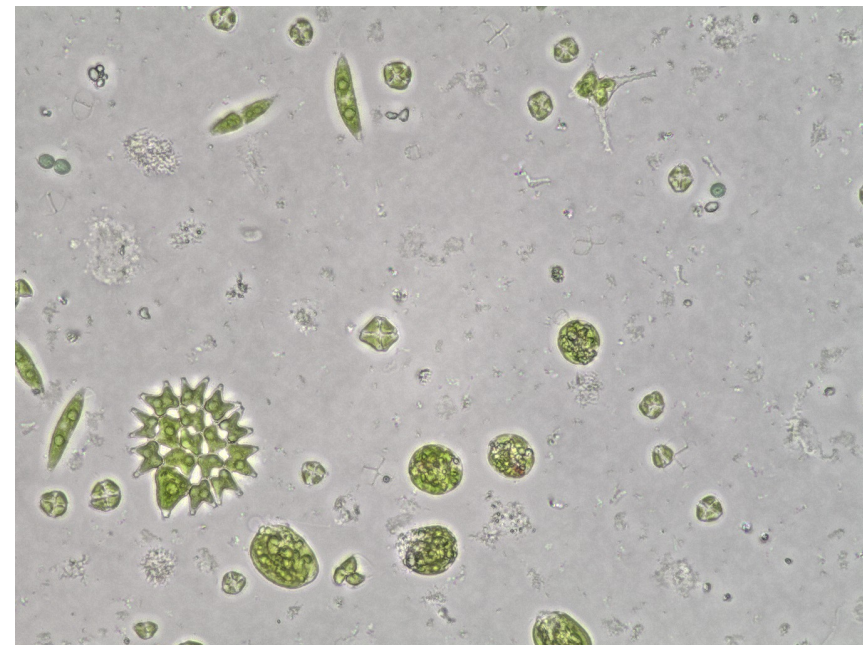

Supplement: Additional file 1 — Comparison of bright field and Quick Full Focus images for the same position. [file 1471-2105-14-115-S1.pdf]
