# Supplementary material for: PlanktoVision – an automated analysis system for the identification of phytoplankton
Source: BMC Bioinformatics. 2013 Mar 27;14:115. doi: 10.1186/1471-2105-14-115 (PMC3636010; doi:10.1186/1471-2105-14-115)
Supplement: Additional file 2 — Description of the used Features in PlanktoVision. [file 1471-2105-14-115-S2.doc]

| Feature | | Description |
| --- | --- | --- |
| Area | | Area of the selection in square pixels |
| Perimeter | The length of the outside boundary of the selection in Pixel | |
| Width & Height | Width and height of the smallest rectangle enclosing the selection. | |
| Major & Minor | Length in pixel of the primary and secondary axis of the best fitting ellipse | |
| Angle | Angle between the primary axis (of the best fitting ellipse) and a line parallel to the x-axis of the image | |
| Circularity | 4π * Area / Perimeter | |
| Aspect Ratio | Major / Minor | |
| Roundness | 4 * Area / (π * Major) | |
| Solidity | Area / Area of the convex hull | |
| Feret's Diameter | The longest distance between any two points along the selection boundary, also known as maximum caliper. | |
| Minimum Feret's Diameter | The shortest distance between any two points along the selection boundary. | |
| Feret angle | Angle between Ferets Diameter and a line parallel to the x-axis of the image. | |
| Integrated Density | The product of area and mean brightness | |
| Skewness | The third order moment about the mean. | |
| Kurtosity | The fourth order moment about the mean. | |
| Saturation | Mean, standard deviation, mode, minimum, maximum pixel value and histogram of the saturation within the Roi. | |
| Color (Hu) | Mean, standard deviation, mode, minimum, maximum pixel value and histogram of Hu within the Roi. | |
| Brightness | Mean, standard deviation, mode, minimum, maximum pixel value and histogram of the brightness within the Roi. | |
| Elliptic fourier descriptor | Fourier descriptors of the closed contour of the Roi according to [21]. | |
| Statistics of the Gray level co-occurrence matrix (glmc) | Angular Second Moment (ASM), Contrast, Correlation Inverse Difference Moment (IDM) and Entropy of the Gray level co-occurence matrix are calculated for the distance of 1 to 5 pixels according to [22, 23]. | |
| Directionality histogram | A 5x5 sobel filter is used to calculate the local gradient in an image. The results are used to derive the local gradient orientation. Based on the orientation a histogram indicating the amount of structures in a given direction is created according to [24]. | |
| Image Moments | Image moments are a particular weighted average of the pixel intensities and are designed to represent different characteristics of the intensity distribution. The used moments are rotation invariant moments based on [25]. | |
| Symmetry measurements | The rotational and reflectional symmetry is calculated based on the outline according to [26]. | |
| Local binary pattern | Feature set that describes the texture inside of the Roi according to [27]. Different patterns are calculated for a radius between 1 and 4. | |
| Phycoerythrin fluorescence | Includes the mean, standard deviation, mode, minimum and maximum value of the fluorescence brightness within the Roi.  Additionally the mean fluorescence intensity is normalized with the background intensity and the percentage of fluorescing parts within the Roi is calculated. | |
| Phycocyanin fluorescence | Features were calculated in the same way as the feature for the phycoerythrin fluorescence. | |
| Chlorophyll fluorescence | | Features were calculated in the same way as the feature for the phycoerythrin fluorescence.  Since particles without chlorophyll showed an unspecific green fluorescence mean, standard deviation, mode, minimum and maximum values within the Roi were also calculated for the red and green channel of the rgb color space as well as for hu of the hsb color space. |
